# High Expression MicroRNA-206 Inhibits the Growth of Tumor Cells in Human Malignant Fibrous Histiocytoma

**DOI:** 10.3389/fcell.2021.751833

**Published:** 2021-11-25

**Authors:** Dejian Li, Kai Zhao, Ziwen Zhao, Bo Jiang, Xianxu Gong, Yan Zhang, Yingqi Guo, Han Xiao, Ye Wang, Hui Liu, Chengqing Yi, Wenguang Gu

**Affiliations:** ^1^Department of Orthopedic Surgery, The First Affiliated Hospital of Harbin Medical University, Harbin, China; ^2^Department of Orthopedics, Shanghai Pudong Hospital, Fudan University Pudong Medical Center, Shanghai, China; ^3^Department of Cardiology, The First Affiliated Hospital of Harbin Medical University, Harbin, China

**Keywords:** miRNA, malignant fibrous histiocytoma (MFH), cancer stem cell (CSC), ALDH^+^ cells, ALDH^–^ cells

## Abstract

**Background:** Malignant fibrous histiocytoma (MFH) is a common type of soft tissue sarcoma and a serious threat to human health. MFH often relapses locally after the curettage is related to the residual cancer stem cells (CSCs). Currently, the dysregulation of microRNA (miRNA) has been found to be closely related to the recurrence of CSCs. However, whether dysregulations of miRNAs exist in MFH, CSCs remained unknown.

**Methods:** In this study, miRNAs in MFH CSCs and MFH common cells were examined by gene probe. Then, target genes and their functions involved in the signal pathway were predicted by the relevant database. Finally, the miRNAs’ target regulatory network was constructed. Furthermore, the miRNAs and target genes were identified by quantitative polymerase chain reaction, whereas miRNA analogs and antagonists were transfected in tumor cells to investigate cell proliferation ability further.

**Results:** Results showed that a total of 47 miRNAs were found, including 16 that were upregulated and 31 that were downregulated. The screened differential miRNA showed a different expression in the cell resistant strains compared with the control group. Quantitative polymerase chain reaction analysis confirmed that the relative abundance of seven miRNAs and four target genes varied significantly. The encouraging issue is that we found Hsa-miR-206 significantly inhibited MFH proliferative activity.

**Conclusion:** Hsa-miR-206 played a key role in regulating MFH CSC properties that might be a representative marker and target for the diagnosis and treatment of MFH in the future.

## Background

Malignant fibrous histiocytoma (MFH) is one of the most common malignant tumors in middle-aged and elderly patients, posing a serious threat to human health. In 1964, O’brien and Stout (1964) first discovered and described MFH, initially named malignant fibrous xanthoma and later renamed MFH. MFH of bone is rare (1–5% of primary bone tumors) and sometimes develops in association with preexisting bone abnormalities (20–25% of MFHs), such as bone infarct (BI) (Gaucher et al., 1991). However, MFH remains a locoregional relapse after traditional therapies, including chemotherapy, radiotherapy, or surgical removal of the tumor tissue. Previous studies have illustrated that these traditional therapies reduced the tumor but left some cancer stem cells (CSCs) behind, which are a unique subset of cells with the potential to self-renew, proliferate indefinitely, and differentiate into common tumor cells (Di et al., 2013; Tan and Zhang, 2016). A previous study has successfully isolated elevated aldehyde dehydrogenase (ALDH) subpopulation cells from the human NMFH-1 cell line to find out the reasons for recurrence in MFH (Molofsky et al., 2004; Li et al., 2015; Mohammed et al., 2016). The result showed that the ALDH^+^ subpopulation exhibits several characteristic CSC properties, including high clonogenicity and self-renewal, increased chemotherapeutic drug resistance, elevated expression of stemness and drug transporter genes, and high tumorigenic potential.

MicroRNAs (miRNAs), which are a kind of endogenous, small, non-coding RNAs, are crucial components of the tumor signaling network that have a close relationship with tumor recurrence (Kong et al., 2012; Cao et al., 2013). Through partial complementation with the 3’-untranslated region (3’UTR) of specific messenger RNAs (mRNAs) and with other regions including 5’UTR and amino acid coding sequence, miRNAs can modulate gene expression by regulating translational efficiency or cleavage of target mRNAs (Pillai, 2005; Lytle et al., 2007; Tay et al., 2008; Takasaki, 2015), thus becoming involved in various physiologically and/or pathologically biological signs of progress (Chen and Rajewsky, 2007). Over the past decade, many different miRNAs have been isolated from different tumors. Among all these miRNAs, different miRNAs played different roles and performed different functions. For example, upregulation of miR-19-3p, miR-21-5p, and miR-221-3p could be identified as potential biomarkers for lung adenocarcinoma diagnosis (Zhou et al., 2017). Downregulated miRNA-34a could promote the capacity of invasion, tumorigenic ability, and self-renewal in human osteosarcoma cells (Zou et al., 2017). Moreover, miRNA-141 and its associated gene FUS have shown the ability to modulate proliferation, migration, and cisplatin chemosensitivity in neuroblastoma cell lines (Wang et al., 2016). However, studies of miRNAs and their functions have rarely been reported in MFH, according to the edge of our best knowledge.

In the present study, we selected differentially expressed miRNAs in the ALDH^+^ and ALDH^–^ subpopulations using gene chip technology that may account for stem cell properties of the ALDH^+^ subpopulation. Subsequently, the related database was used to predict the target genes, their function, and signaling pathway. The miRNA targeting network was then constructed according to the signaling pathway related to target genes. Quantitative polymerase chain reaction (qPCR) was used to quantify core miRNA and target genes. The effect of miRNAs on tumor cells behavior, cell proliferation, and drug resistance were also investigated. Results showed that high expression miRNA-206 could inhibit the growth of tumor cells in human MFH effectively. Our findings may contribute to establishing diagnosis and treatment for MFH.

## Materials and Methods

### Authentication of Cell Lines

The human NMFH-1 cell line was obtained from Niigata University Graduate School of Medical and Dental Sciences. This cell line was established in 2005 and analyzed with spectral karyotyping and comparative genomic hybridization. Specific verification methods and analysis are shown in Li et al. (2015). Cells were cultured in RPMI1640 supplemented with 10% fetal bovine serum in a humidified atmosphere containing 5% CO_2_ atmosphere at 37°C.

### Array Chip Information

Exiqon’s miRNA array chip features Tm-normalized LNA-enhanced capture probes, designed for excellent specificity and sensitivity even for AT-rich miRNAs. In addition, they offer great reproducibility with a 99% correlation between arrays and a dynamic range greater than five orders of magnitude. The seventh generation of miRCURY^TM^ LNA Array (v.18.0) (Exiqon) contains 3,100 capture probes, covering all human, mouse, and rat miRNAs annotated in miRBase 18.0, as well as all viral miRNAs related to these species. In addition, this array contains capture probes for 25 miRPlus human miRNAs.

### Experimental Grouping

Six Exiqon miRNA array chip data were divided into two groups: the experimental group was extracted from stem cells (three samples labeled ALDH^+^1, ALDH^+^2, and ALDH^+^3); for the control group, the flow separation from the stem cell residual was used (three samples labeled ALDH^–^1, ALDH^–^2, and ALDH^–^3). For the differential miRNA validation experiment, the experiment was divided into two groups (ALDH^+^ group and ALDH^–^ group), each of three samples. For the cell drug resistance experiment, the experiment was divided into three groups (DCTX, GCTB, and control), each of three samples. For the cell proliferation array, cells were divided into five groups, including Mimic-NC, Inhibitor-NC, Hsa-miRNA-mimic, Hsa-miRNA-Inhibitor, and blank control (B).

### Screening Differentially Expressed MicroRNA

During the chip detection process, a random variance model (RVM) modified *T*-test was used to compare the two sample groups. Each group had three chips belonging to the small sample data (less than 30). The miRNA difference was measured by calculating the miRNA significance level (*P*-value) and the false-positive rate (FDR) in each group, using the Two-Class Dif tool (Wright and Simon, 2003; Yang et al., 2005; Clarke et al., 2008). RVM *t*-test was applied to filter the differentially expressed genes for the ALDH^+^ and ALDH^–^ groups because it can raise degrees of freedom effectively in the cases of small samples. After the significant analysis and FDR analysis, we selected the differentially expressed genes according to the *p*-value threshold. *P*-value < 0.05 was considered as a significant difference.

### Target Gene Prediction

When studying the biological function and mechanism of miRNA, it is important to identify miRNA target genes accurately. MiRNA, which is essential for target mRNA degradation or inhibition by combining RNA-induced silencing complex in 3’UTR of miRNA, is very relevant to the cell biological behavior, cell proliferation, differentiation, development, and apoptosis. At present, bioinformatics analysis is used to predict target genes. In this study, we used TargetScanHuman 7.2 to predict miRNA target genes.

### Target Functional Analysis of Target Genes

Gene Ontology (GO) database is a cross-species, comprehensive, descriptive platform used to describe the gene and protein function in a tree-like manner and the hierarchical relationship between gene functions. In this study, GO was performed for the predicted target genes and their function. The *P*-value and FDR of each gene function were calculated by Fisher exact test and multiple comparison test. *P* < 0.001 was used as the standard to select the low error rate, targeting, and significant target gene function.

### Analysis of Signal Transduction Pathways Involved in Target Genes

The signal pathway analysis was analyzed based on the Kyoto Encyclopedia of Genes and Genomes database. The Kyoto Encyclopedia of Genes and Genomes (Kanehisa et al., 2004; Affara et al., 2013) is a database used to analyze intergenic relationships, gene functions, and genomic information. Target genes involved in the pathway were analyzed by the Fisher exact test and chi-square test. A significant pathway was obtained according to *P*-value < 0.001.

### Network Construction Based on Target Gene Pathway Analysis

This method combines the gene pathway database and the target sequence analysis technique to establish a differential miRNA pathway network using the regulatory relationship between the differential miRNA and the target gene signaling pathway. At the same time, graph theory is used to evaluate the control status of miRNA and pathway in the network by degree. According to the values of degree in graph theory, the key miRNA and crucial pathway were obtained.

### Quantitative Polymerase Chain Reaction

Total RNA was extracted from both the ALDH^+^ and ALDH^–^ cells by the Trizol method. Reverse transcription of common genes was detected by reverse transcription kit (TaKaRa, #RR036A). Reverse transcription of miRNA was detected using special kits (TIANGEN KR201): miRNA 3’ends were treated with Poly (A) and were consequently transcribed into complementary DNA (cDNA) by reverse transcription reaction. Gene expression was analyzed according to the qPCR kit (Invitrogen, #4367659): 2 × SYBR green mix buffer 10 μl; forward primer 0.4 μl; reverse primer 0.4 μl; cDNA solution 2 μl; RNase free dH_2_O up to 20 μl. qPCR was performed in 96-well microtiter plates (Thermo Fisher Scientific, United States) using the ABI ViiA7 Fluorescence qPCR instrument. Amplifications were performed using a 10-min enzyme activation at 95°C, followed by 40 cycles of denaturation at 95°C for 15 s, and then with annealing/extension at 60°C for 60 s. At the end of each run, a melting curve analysis was performed from 60 to 95°C. All samples and negative controls were amplified in triplicate, and the obtained mean value was then used for further analysis. A cycle of quantification (Cq) values of > 35 was excluded from further mathematical calculations. A “no template sample” (RNA from reverse transcription without reverse transcriptase) and a sample without RNA or cDNA were the negative controls. The primer sequences used for amplification are listed in Supplementary Table 1. Using the two sets of data to calculate the mean and standard deviation to draw the histogram, the difference was statistically analyzed (*P* ≤ 0.05).

### MicroRNA and Mechanism of Drug Resistance

In this experiment, NMFH-1 cells were treated with two different drugs, docetaxel (Selleck S148) and gemcitabine (Selleck S1714). Briefly, cells were collected, centrifuged, washed with 3-ml D-Hank’s solution, and counted. 3 × 10^5^ cells/ml were then plated in a 24-well plate and consequently treated with 50-mM gemcitabine or 10-mM docetaxel dissolved in DMSO. The concentrations of 10-nM gemcitabine and 0.01-nM docetaxel were set as a blank control. After treating cells with different drugs for 24 h, cell viability was investigated, and a production concentration curve was constructed. Briefly, docetaxel IC50 of NMFH-1 cells was 36 nM, whereas gemcitabine IC50 was 41 μM; after treating cells with 36-nM docetaxel and 41-μM gemcitabine, high cell death was observed; therefore, the concentrations were reduced to 9 nM and 20 μM, respectively. Consequently, according to the drug IC50 of NMFH-1, an appropriate amount of docetaxel or gemcitabine was added to the complete medium of NMFH-1 cells for 1 month. One to two times a week, dead cells were removed in the process of resistance to domestication. Resistant strains were detected by PCR gene quantification. RNA extraction electrophoresis shows: the bands of 28s rRNA, 18s rRNA are clearly visible. This indicates that the RNA is not degraded. The optical density (OD) value of A260/A280 was 1.9–2.2 (no pollution in the process of extracting protein was observed).

### Cell Proliferation Test

To examine changes in cell proliferation, mimics and antagonists from seven miRNAs were used to transfect tumor cells. Briefly, NMFH-1 cells were cultured in a 6-cm dish until 80–90% confluence. Cells were then washed two times with D-Hank’s solution, trypsinized, and counted. Then, cells were plated into a 24-well plate (2.5 × 10^5^ cells/well) and cultured at 37°C 5% CO_2_ for 24 h. Consequently, each well was incubated with 400-μl fresh medium and 100-μl Opti-MEM I, 40 pmol of miRNA, and 1-μl siRNA-mate for 4–6 h. Mimics and antagonists of miRNA were successfully transfected into tumor cells through Lipofectamin2000 biological techniques. When 40-pmol Lipofectamine 2000 was transfected with 2-μl NC-FAM, the transfection efficiency was approximately 40%, which was relatively high.

To measure cell viability, cells were incubated with 100-μl CCK-8 reagent/well (diluted 1:10 with the medium) for 2 h, after which the absorbance was detected at 490 nm (0-h time point). Consequently, the cell medium was replaced, and cells were incubated for an additional 48, 72, 96, and 120 h; the absorbance was measured after each time point. In the cell proliferation test, maximum and minimum values of five repetitions in each sample were removed, and only the average of three replicates was taken to reduce the data deviation. To reduce the influence of the CCK-8 reagent and the different culture times on the numerical value of each time point, a zero adjustment hole was used. When collating data, the three complex holes were subtracted from the value of zero hole.

### Statistical Analysis

SPSS22.0 package analysis was used in this part, the selection of differential mRNA was analyzed by *T*-test with RVM, and the difference was defined statistically significant if the *p*-value < 0.05. GO analysis was performed by Fisher exact test and multiple comparison test, and *p*-value < 0.001 was considered statistically significant. The pathway analysis was done using the Fisher exact test and chi-square test, and *p*-value < 0.001 was considered statistically significant. Real-time PCR and cell invasion experiments were performed by *t*-test with correlated samples where *p*-value < 0.05 was considered statistically significant. The Kolmogorov–Smirnov test was used to verify the normal distribution. Single-factor analysis of variance (one-way analysis of variance) was used for drug resistance and cell proliferation test where *p*-value < 0.05 was considered statistically significant.

## Results

### Screening ALDH^+^ and ALDH^–^ Cells for Differentially Expressed MicroRNAs

**FIGURE 1:**
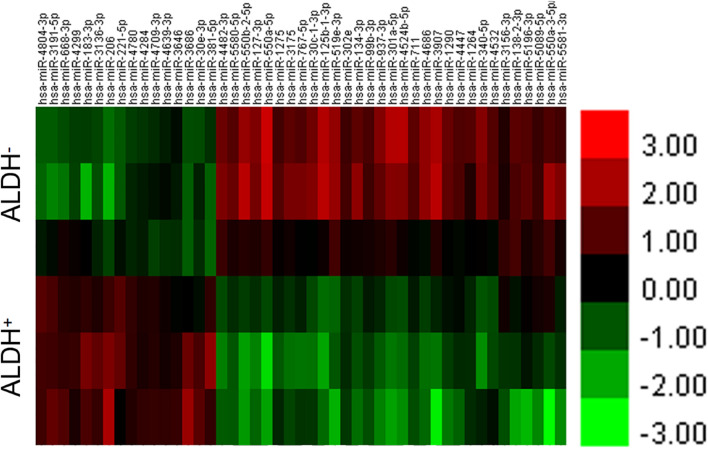
Differential miRNA for cluster analysis. Abscissa represents name of sample between groups, ordinate represents differential gene, red represents differential gene in grouped sample expression value is high, and green represents differential gene in sub. Expression in sample was low.

The difference between the experimental group (ALDH^+^ cell group) and the control group (ALDH^–^ cell group) was analyzed using a *t*-test of the RVM. A total of 47 miRNAs were found, including 16 that were upregulated (Supplementary Table 2A) and 31 downregulated (Supplementary Table 2B). At the same time, according to the results discussed earlier, cluster analysis was carried out to get the dendrogram (Figure 1).

### Prediction of Target Genes and Their Function

**FIGURE 2:**
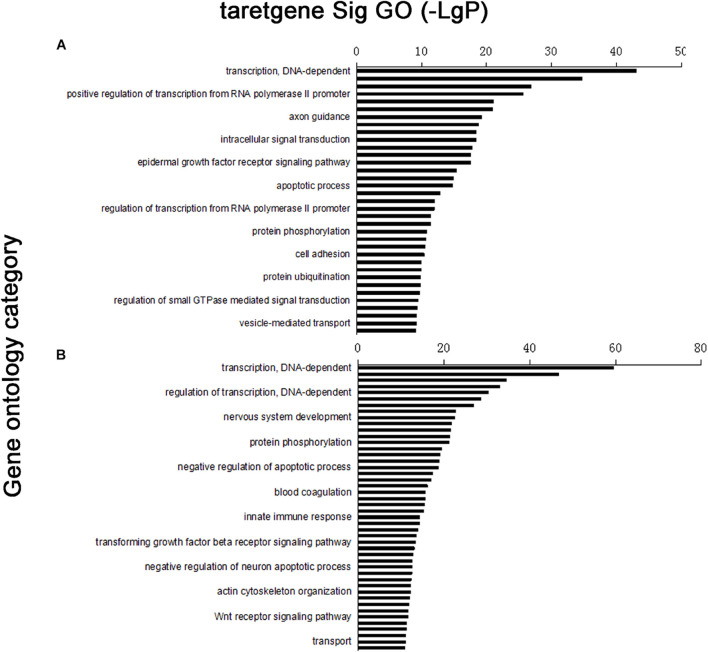
Distribution map of significant gene functions: vertical coordinate was function of target gene, horizontal coordinate was enrichment. Each column in graph represented a significant function of target gene. **(A)** Upregulated miRNA target gene enrichment partly; **(B)** downregulated miRNA target gene enrichment partly.

Four thousand five hundred eighty-nine target genes were obtained by taking the intersection between the two databases; 6,870 target genes were predicted by TargetScan, whereas 13,563 target genes were predicted by the miRanda database. The number of target genes of upregulated and downregulated miRNAs is partly shown in Supplementary Tables 3A,B. Furthermore, target gene function was obtained by the GO database, which had 204 significant gene functions based on upregulated miRNAs and had 320 based on downregulated miRNAs. Transcription DNA-dependent, regulation of transcription DNA-dependent, signal transduction, positive regulation of transcription from RNA polymerase II promoter, and small molecule metabolic process were the most significant features in the target gene function of upregulated miRNAs (Supplementary Table 4). The target gene function of downregulated miRNAs has some most significant features, including transcription DNA-dependent, regulation of transcription DNA-dependent, small molecule metabolic process, positive regulation of transcription from RNA polymerase II promoter, signal transduction positive regulation of transcription, DNA-dependent, etc. (Supplementary Table 4). According to the significance function of the target gene of upregulation and downregulation of miRNA, the targeting maps can be made for the function of significance by enrichment degree in the database (Figure 2).

### Signal Pathway Analysis

**FIGURE 3:**
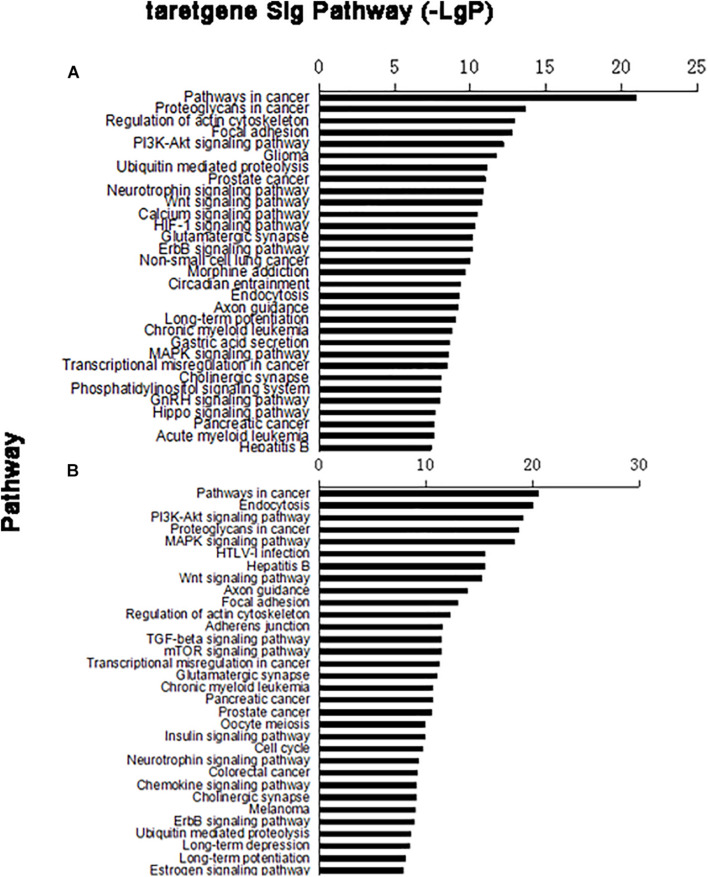
Column chart of signal pathways. Vertical coordinate was signal pathway; horizontal coordinate was negative logarithm (−LgP) of *p*-value. **(A)** Upregulated miRNA target gene pathway enrichment partly; **(B)** downregulated miRNA target gene pathway enrichment partly.

In the target gene of upregulated miRNA, 79 distinct signaling pathways were selected; in the target gene of downregulated miRNA, 95 distinct signaling pathways were selected. Based on the predicted levels of the signaling pathway significance, a histogram of the upregulated miRNA predicted signal pathway (Figure 3A) and the downregulated miRNA predicted signal pathway were constructed (Figure 3B).

### MicroRNA Pathway Network

**FIGURE 4:**
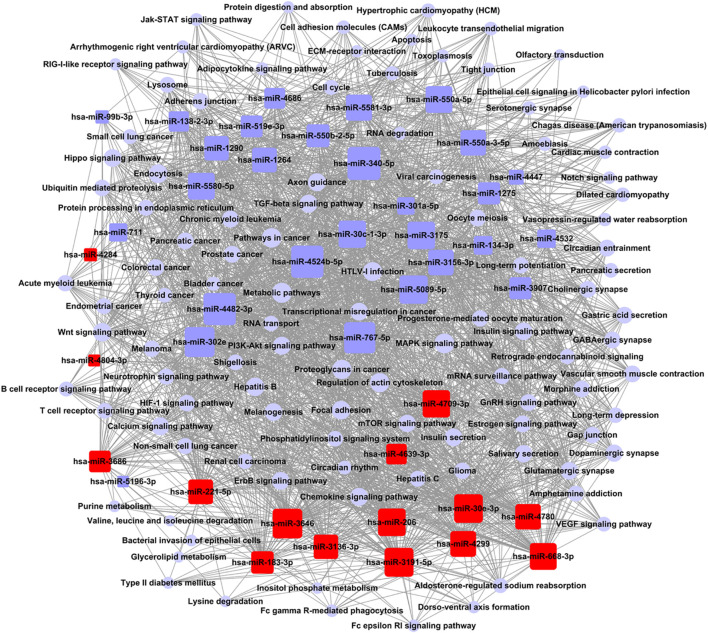
Network control chart of miRNAs and signal pathways. Rounded rectangle indicated miRNAs (red indicated an increase; blue indicated down), blue purple circle indicated signal pathway, and straight line represented regulation relationship between them. Evaluation of regulation status was judged by degree, which was contribution of one miRNA to surrounding pathways. More miRNAs regulated pathways, bigger their areas. More pathways received regulation of miRNAs, larger their areas. Key miRNA and pathway were largest in network.

According to the relationship between miRNAs and the signal pathways of a target gene, the targeting regulatory network was set up (Figure 4). According to the values of degree in graph theory, the key miRNA and crucial pathway were obtained. The key miRNA in the network was hsa-miR-340-5p (Supplementary Table 5A). The critical crucial pathway was metabolic pathways (Supplementary Table 5B).

### Quantitative Polymerase Chain Reaction

**FIGURE 5:**
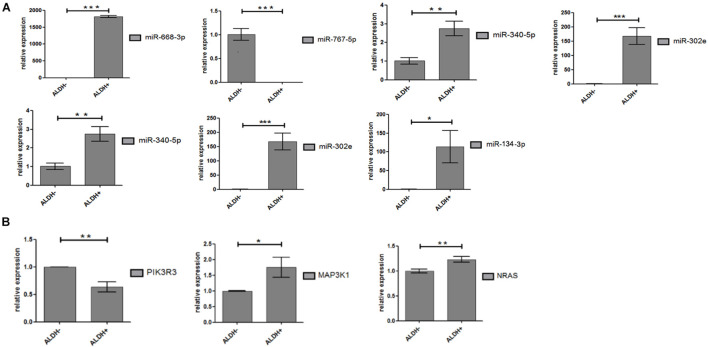
Differential expression of miRNA and target genes. **(A)** Differential expression of miRNA between ALDH + cells and ALDH- cells (three samples of each group). Difference was statistically significant. **(B)** Differential expression of target gene between ALDH + cells and ALDH- cells (three samples of each group). Difference of target (PIK3R3, AKT3, and MAP3K1) was statistically significant (means ± SD; *P ≤ 0.05, **P ≤ 0.01, and ***P ≤ 0.001).

Seven miRNAs (hsa-miR-340-5p; hsa-miR-767-5p; hsa-miR-302e; hsa-miR-206; hsa-miR-668-3p; hsa-miR-134-3p; and hsa-miR-487b-3p) and 12 target genes were validated by qPCR. Hsa-miR-134-3p and hsa-miR-487b-3p were not included in the results of gene chip screening and were included in the verification standard. That is because hsa-miR-134-3p, hsa-miR-487b-3p, and hsa-miR-668-3p belong to the same miRNA family and are located on chromosome 14 long arm 14q32 region, with similar biological functions (O’brien and Stout, 1964; Gaucher et al., 1991). Furthermore, experimental statistics showed that seven differentially expressed miRNAs and three target genes (MAP3K1, PIK3R3, and NRAS) were statistically significant (Figures 5A,B). As we expected, the results show that miRNAs have significant differences between ALDH^+^ and ALDH^–^ cells.

### MicroRNA and Drug Resistance Mechanism

**FIGURE 6:**
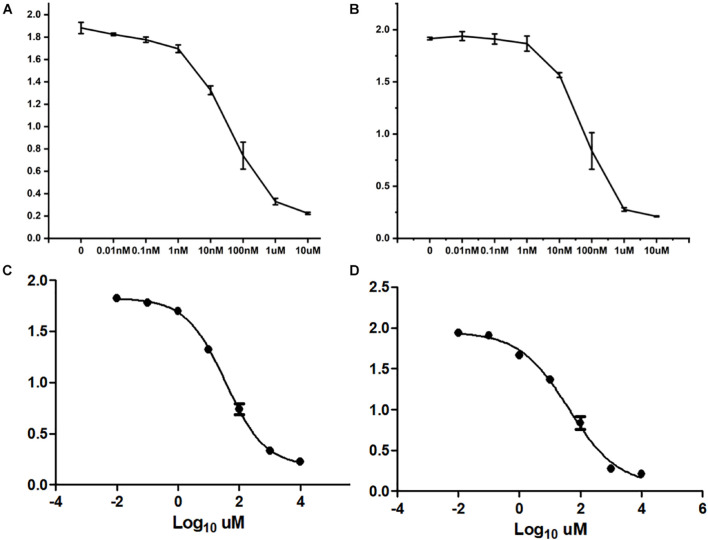
Half-maximal inhibitory concentration. **(A)** Curve of cell growth, which was cultured in different concentrations of docetaxel. **(B)** IC50 of docetaxel. **(C)** Curve of cell growth, which was cultured in different concentrations of gemcitabine. **(D)** IC50 of gemcitabine.

**FIGURE 7:**
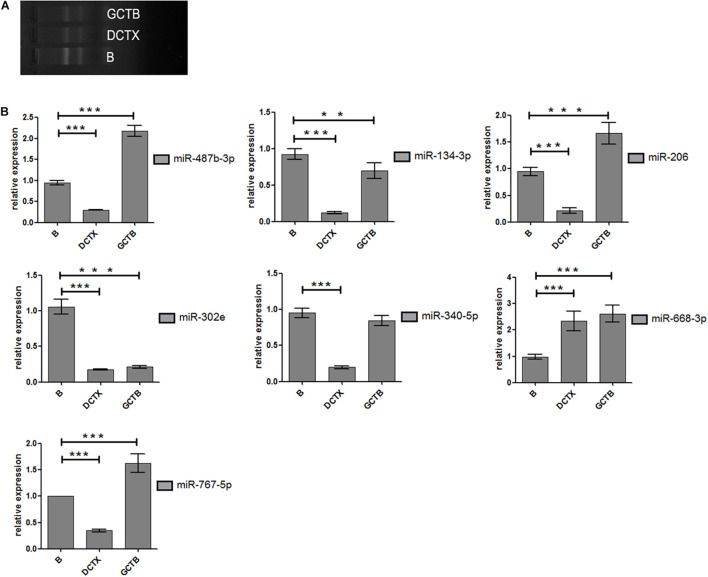
Experimental results of cell proliferation. **(A)** Electrophoresis diagram of RNA extraction. **(B)** Statistical analysis of miRNA-206 (means ±SD; one-way analysis of variance **P ≤ 0.01 and ***P ≤ 0.001, which has statistical significance). miR-206-inhibitor vs. NC-inhibitor; miR-206-inhibitor vs. miR-206mimics; miR-206-inhibitor vs. blank. *P* ≤ 0.05 Results of data are statistically significant, which indicate ability of miRNA-206 to inhibit cell proliferation.

After treating cells with different drugs for 24 h, cell viability was investigated, and a production concentration curve was constructed (Figures 6A,B). Briefly, docetaxel IC50 of NMFH-1 cells was 36 nM, whereas gemcitabine IC50 was 41 μM (Figures 6C,D); after treating cells with 36-nM docetaxel and 41-μM gemcitabine, high cell death was observed; therefore, the concentrations were reduced to 9 nM and 20 μM, respectively. Resistant strains were detected by PCR gene quantification. RNA extraction electrophoresis shows: the bands of 28s rRNA and 18s rRNA are clearly visible. This indicates that the RNA is not degraded (Figure 7). The OD value of A260/A280 was 1.9–2.2 (no pollution in the process of extracting protein was observed). The results showed that Hsa-miR-668-3p was highly expressed in docetaxel-resistant strains, whereas other six miRNAs (hsa-miR-134-3p, hsa-miR-206, hsa-miR-302e, hsa-miR-340-5p, hsa-miR-487b-3p, and hsa-miR-767-5p) had low expression. Nevertheless, hsa-miR-206, hsa-miR-487b-3p, hsa-miR-668-3p, hsa-miR-767-5p were highly expressed in gemcitabine-resistant strains, whereas hsa-miR-134-3p, hsa-miR-302e, and hsa-miR-340-5p were low (Figure 7). Furthermore, no statistical difference in miRNA-340-5p was found in gemcitabine-resistant strains compared with the control group, whereas a significant difference was observed in other miRNAs.

### MiRNA Transfection Cell Invasion Results

**FIGURE 8:**
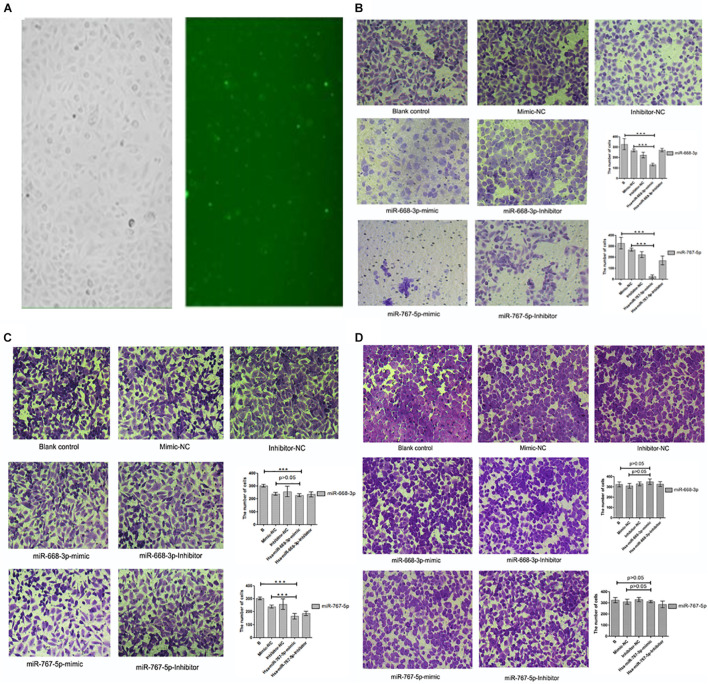
Results of cell invasion test. **(A)** Cell transfection results of drugs. **(B)** Results and statistical analysis of first cell invasion test (miRNA-767-5P and miRNA-668-3p were able to inhibit cell invasion and metastasis). **(C)** Results and statistical analysis of second cell invasion test (miRNA-767-5P was able to inhibit cell invasion and metastasis). **(D)** Results and statistical analysis of third cell invasion test (miRNA-767-5P and miRNA-668-3p were not able to inhibit cell invasion and metastasis) (means ± SD; ***P ≤ 0.001, which has statistical significance).

In this experiment, three repeated experiments were conducted to obtain different results. We transfected mimics and antagonists into tumor cells through biological techniques. The transfection results are shown in Figure 8A. In the first experiment, we found that Hsa-miR-668-3p, Hsa-miR-767-5p mimics, and antagonists were significantly lower than that of the control group on the basis of cell counts, and the statistical analysis was statistically significant (*P* < 0.05). To verify the first result, we carried out second transfection experiments on Hsa-miR-668-3p and Hsa-miR-767-5p. The results showed that the effects of Hsa-miR-668-3p and Hsa-miR-767-5p were significantly reduced, and the statistical analysis showed only Hsa-miR-767-5p significance. In the third experiment, we found that both 668 and 767 had no effect on cell invasion. Photographic analysis results and cell count are shown in Figures 8B–D.

### MiRNA Transfection Cell Proliferation Results

**FIGURE 9:**
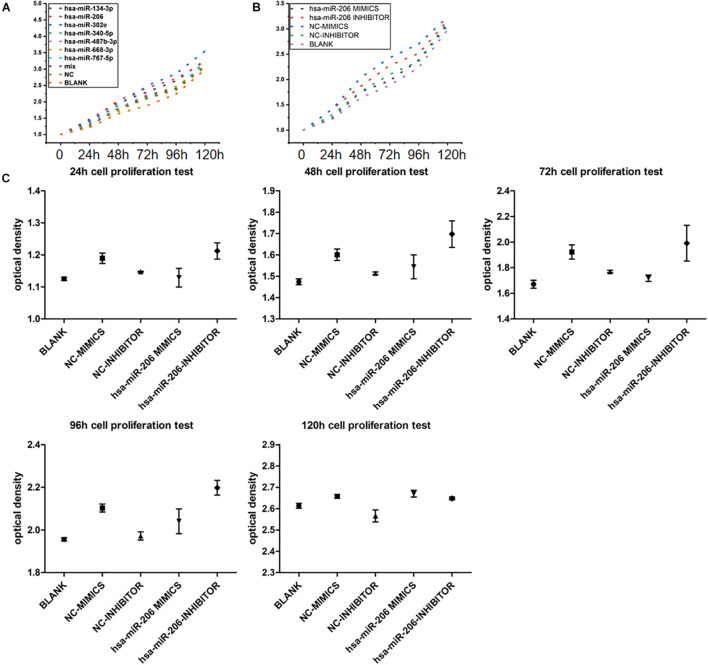
Experimental results of cell proliferation. **(A)** Proliferative curve of miRNA at different times. **(B,C)** Statistical analysis of miRNA-206 at different times (means ± SD; one-way analysis of variance; *P* ≤ 0.05, MiRNA-206 was not statistically significant in 120 h).

In the cell proliferation test, when we grouped the data, the maximum and minimum values of five repetitions in each sample were removed, and only the average of three replicates was taken to reduce the data deviation. To avoid the addition of CCK-8 reagent, the difference in culture time has an effect on the value of each time point, setting the zero hole. When the data are sorted, the above three holes are subtracted from the zero value. The average of each group of samples at 0, 24, 48, 72, 96, and 120 h are showed in Figures 9A,B. Statistical analysis showed that miRNAs were statistically significant at 24, 48, 72, 96, and 120 h (Figure 9C). The results showed that miRNA-206 significantly inhibited cell proliferation.

## Discussion

Current clinical parameter imaging and laboratory analytic criteria are insufficient for establishing a diagnosis and treatment for NMFHs, a high-grade and aggressive sarcoma, most commonly located in the extremities and retroperitoneum (O’brien and Stout, 1964). With the development of tumor stem cell theory, many researches have been focusing on extracting stem cells that provided a new theoretical basis for the treatment of tumors (Li et al., 2015; Yu et al., 2016). However, CSC in gene therapy, also known as tumor markers, is still worthwhile investigating.

According to the result of gene chip and qPCR, miRNA showed different differences between ALDH^+^ cells and ALDH^–^ cells in NMFH-1. We believe that the differential expression of miRNAs in ALDH^+^ cells may be strongly linked to the nature of their stem cells. Previously, many experiments have proved that miRNAs play a significant role in the development of tumors and thus cause changes in the biological characteristics of tumors. For example, Wang et al. (2015) have researched pancreatic cyst fluid miRNAs from low-grade benign and high-grade invasive lesions using next-generation sequencing and has found many meaningful miRNAs that cause high invasiveness in benign tumors. Meantime, the miRNAs (miR-340-5p, miR-767-5p, miR-302e, miR-206, miR-668-3p, miR-134-3p, and miR-487b-3p) we validated are also reported in other studies. MiRNA302e can induce the production of pluripotent stem cells (Zhang and Wu, 2013); miRNA-206 can inhibit the development of breast cancer (O’Day and Lal, 2010); miRNA-134 can inhibit lung cancer cells by targeting the epidermal growth factor receptor (Qin et al., 2016); miRNA-340 inhibits tumor cell proliferation and induces apoptosis in endometrial carcinoma cell line (Xie et al., 2016). If genes determine a biological trait, when a biological trait of a cell or individual changes, its internal genes are bound to change too. Target genes achieve the biological effects of miRNAs. Through our experiments, we found three target genes (MAP3K1, PIK3R3, and NRAS) that were significantly different. According to the target regulation network of miRNA, we can conclude that MAP3K1 is the target gene for miRNA-206, miRNA-340-5p, and miR-5581-3p; PIK3R3 is the target gene for miRNA-206 and hsa-miR-4299; NRAS is the target gene for miR-30e-3p and miRNA-767-5p; MAP3K1 is a member of the family of mitogen-activated protein kinases that regulates apoptosis, survival, migration, differentiation, and other multiple effects of cells (Cuevas et al., 2006; Gallagher, 2011). PIK3R3 participates in the regulation of all kinds of tumors and diseases by interacting with the insulin-like growth factor 1 receptor, IRS1 and retinoblastoma protein (Chen et al., 2016; Hanyuda et al., 2016). NRAS is a member of the RAS gene family. RAS gene changes are mainly point mutation and gene amplification, resulting in Ras-GTP sustained activation and causing malignant proliferation and metastasis. It is involved in a wide variety of biological processes, including cell proliferation, differentiation, apoptosis, tumorigenesis, as well as glycogen synthesis and glucose uptake (Turner et al., 2015; Kim et al., 2016; Stottrup et al., 2016). The present findings suggest that differential miRNA may be related to stem cell properties of ALDH^+^ cells in MFH by acting on their target genes.

To better understand the effect of miRNA on ALDH^+^ malignant fibrous tissue cells, we performed biological function experiments of miRNA, and we found that differential expression in miRNA was related to cell drug resistance. Because of the different mechanisms of drugs act on the tumor, the expression of miRNA was different in the two cell drug-resistant strains. Experimental results showed that Hsa-miR-668-3p was highly expressed in docetaxel-resistant strains, whereas six miRNAs (hsa-miR-134-3p, hsa-miR-206, hsa-miR-302e, hsa-miR-340-5p, hsa-miR-487b-3p, and hsa-miR-767-5p) had low expression. In contrast, hsa-miR-206, hsa-miR-487b-3p, hsa-miR-668-3p, and hsa-miR-767-5p were highly expressed in gemcitabine-resistant strains, whereas hsa-miR-134-3p, hsa-miR-302e, and hsa-miR-340-5p were low expression compared with the control group. These results were statistically significant, and they implied that tumor drug resistance might be caused by the overexpression and suppression of these differential miRNAs. Meanwhile, these results further illustrate the importance and necessity of our screening of miRNAs in the ALDH^+^ cell for the study of this tumor. In the present study, MiR-767 has been found to promote cell proliferation in human melanoma by suppressing CYLD expression (Zhang and Guo, 2018). Zhuang et al. (2017) has proved that the dysregulated lncRNA-LET/NF90/miR-145 axis by gemcitabine-induced TGF-β1 promotes UBC chemoresistance through enhancement of cancer cell stemness. Therefore, the discovery of which specific miRNA or which group was essential, which could provide new solutions for clinical tumor diagnosis and gene therapy. Due to the ALDH^+^ cells that have the characteristics of stem cells, we think that the biological trait of drug resistance may be closely related to the regulation disorder of miRNAs.

In cell proliferation assays, we found that miRNA-206 significantly inhibited tumor cell growth in the differential miRNAs. According to the theory of CSCs, the reason why tumors are difficult to eradicate is the existence of CSCs. CSCs are a unique subset of cells with the potential to self-renew, indefinitely proliferate, and differentiate into common tumor cells. In the early stage, our team has demonstrated that ALDH^+^ cells have the characteristics of stem cells. The experimental results indicate that miRNA-206 may act on ALDH^+^ cells and change the proliferation characteristics of cancer cells. Therefore, we believe that miRNA-206 might be used as a biomarker for the diagnosis of CSCs (ALDH^+^ cell) and as a therapeutic target to achieve the purpose of tumor eradication. The present research reports that miRNA-1 and miRNA-206 regulate skeletal muscle satellite cell proliferation and differentiation by repressing Pax7 (Chen et al., 2010). MiR-1/206 is also known to target the hepatocyte growth factor/scatter factor receptor MET and regulates the development of liver cancer and lung cancer (Datta et al., 2008; Nasser et al., 2018; Yi et al., 2019). Moreover, it has been shown that miR-206, previously viewed as a muscle-specific miRNA, is a key regulator in this process that regulates the mechanism of osteoblast differentiation (Wang et al., 2018). Hence, we can learn that miRNA-206 plays a very important role in normal cell function. Meanwhile, miRNA-206 plays a critical role in many diseases, such as CCL2 as a direct target of miR-206, and the upregulation of CCL2 caused by the downregulation of miR-206 was responsible for the development of severe HEV71 encephalitis (Zhang et al., 2017). A study by Xing et al. (2017) has proved that miR-206 was able to substantially suppress the proliferation of vascular smooth muscle cells *via* targeting forkhead box protein subfamily P1 (FOXP1). MiR-206 has also directly targeted the oncogenes KRAS and annexin a2 (ANXA2), acting as a tumor suppressor in various types of cancer by blocking cell cycle progression, cell proliferation, migration, and invasion. Therefore, according to the current research and our experimental results, we suggest that miRNA-206 may inhibit cell proliferation in MFH by a certain regulatory mechanism that miRNA can partially combine with the 3′UTR of specific mRNAs and with other regions, including 5′UTR and amino acid coding sequence. MiRNA-206 presents a unique opportunity for the development of biomarkers of ALDH^+^ cells that would allow early detection of lesions and elimination of tumors. However, our study has some limitations; in our experiment, we only used six microarray genes. Also, the specific mechanism of action of miRNA in animal models needs further study.

## Conclusion

In conclusion, this study screened differential miRNA in MFH and predicted target genes and signaling pathways involved in miRNA. At the same time, through the biological function of miRNA, we confirmed that hsa-miR-206 are crucial to the inhibition of NMFH cells and might be biomarkers for the diagnosis and treatment of this tumor. The reported results can provide us with a preliminary understanding of the differential expression of miRNAs in the ALDH^+^ cells with the presence of CSC in MFHs, which in turn can be useful in establishing the diagnosis and treatment for CSCs. MiRNA expression could also be potentially relevant for the development of clinical treatments.

## Data Availability Statement

The data presented in the study are deposited in the figshare repository, accession number doi: 10.6084/m9.figshare.16815334.

## Author Contributions

WG, CY, and HL designed the study. DL, KZ, ZZ, BJ, XG, YZ, YG, HX, and YW were involved in cell culture, gene chip production, and cell biology experiments. DL, KZ, and ZZ were involved in the construction of the target control network and the analysis of the related data results. DL wrote the manuscript with help from all the authors. All authors read and approved the final form of the manuscript.

## Conflict of Interest

The authors declare that the research was conducted in the absence of any commercial or financial relationships that could be construed as a potential conflict of interest.

## Publisher’s Note

All claims expressed in this article are solely those of the authors and do not necessarily represent those of their affiliated organizations, or those of the publisher, the editors and the reviewers. Any product that may be evaluated in this article, or claim that may be made by its manufacturer, is not guaranteed or endorsed by the publisher.

## Abbreviations

MFH: malignant fibrous histiocytoma
ALDH: aldehyde dehydrogenase
CSC: cancer stem cell
GO: Gene Ontology
miRNA: microRNA
RNA: ribonucleic acid
UTR: untranslated region
CCK-8: cell counting kit-8
DCTX: Docetaxel
GCTB: Gemcitabine.
