# Supplementary material for: High Expression MicroRNA-206 Inhibits the Growth of Tumor Cells in Human Malignant Fibrous Histiocytoma
Source: Front Cell Dev Biol. 2021 Nov 25;9:751833. doi: 10.3389/fcell.2021.751833 (PMC8656228; doi:10.3389/fcell.2021.751833)
Supplement: Supplementary file 1 [file Table_1.DOC]

Table I：The primer sequences used for amplification

| Target gene | Primer sequences |
| --- | --- |
| MAPK1-RT | F：5'-CTGCTCAACACCACCTGTGATCTCA-3' |
| MAPK1-RT | R：5'-AATTTCTGGAGCCCTGTACCAACGT-3' |
| AKT1-RT-F | F：5'-TCAAGAAGCAGGAGGAGGAGGAGAT-3' |
| AKT1-RT-R | R：5'-CAGGTACTCAAACTCGTTCATGGTC-3' |
| AKT3-RT-F | F：5'-AGGAAAGGGAAGAATGGACAGAAGC-3' |
| AKT3-RT-R | R：5'-GGCATCCATCTCTTCCTCTCCTATA-3' |
| MAP2K1-RT-F | F：5'-TGGATGGAGGTTCTCTGGATCAAGT-3' |
| MAP2K1-RT-R | R：5'-TCTTGTGCTTCTCCCTCAGATATGT-3' |
| NRAS-RT-F | F：5'-ACAAGTGTGATTTGCCAACAAGGAC-3' |
| NRAS-RT-R | R：5'-TGTAAAAAGCATCTTCAACACCCTG-3' |
| PIK3R1-RT-F | F：5'-CGCCTCTTCTTATCAAGCTCGTGGA-3' |
| PIK3R1-RT-R | R：5'-TTCCAAGTCCACGGAGGGTGTATCA-3' |
| PIK3R3-RT-F | F：5'-GGGAGGTGATGATGCCCTATTCGA-3' |
| PIK3R3-RT-R | R：5'-ACTGCTGAAGTCATTGGCTTAGGTG-3' |
| RHOA-RT-F | F：5'-GGTGAAACCTGAAGAAGGCAGAGAT-3' |
| RHOA-RT-R | R：5'-GCCATTTCAAAAACCTCTCTCACTC-3' |
| MAPK9-RT-F | F：5'-CCTGAAGATCCTTGACTTTGGCCTG-3' |
| MAPK9-RT-R | R：5'-TAGCCCATACCCAGGATGACTTCGG-3' |
| MAP3K1-RT-F | F：5'-CAAAGGGTTGCACAAGATGGATGAT-3' |
| MAP3K1-RT-R | R：5'-CGCCTATTTCTCCTTTCCAACCATT-3' |
| PTEN-RT-F | F：5'-GACGAACTGGTGTAATGATATGTGC -3' |
| PTEN-RT-R | R：5'-ATACACATAGCGCCTCTGACTGGGA -3' |
| MAGI2-RT-F | F：5'-AAGTGGTGCTCTCCTAGAAAGTGGG-3' |
| MAGI2-RT-R | R：5'-GGCTCCTGGAAGTATCTGGTCTGTT-3' |
| HmiR-134-3p-FO | F：5'-ATTGATACCTGTGGGCCACCTA-3' |
| HmiR-134-3p -RE | R：5'-TATGGTTGTTCACGACTCCTTCAC-3' |
| HmiR-206-FO-3 | F：5'-CTGCCGTGGAATGTAAGGAA-3' |
| HmiR-206-RE-3 | R：5'-TATGGTTGTTCTGCTCTCTGTCTC-3' |
| HmiR-302e-FO | F：5'-ATTCGCAGTGCTGTAAGTGCTTC-3' |
| HmiR-302e -RE | R：5'-TATGGTTGTTCACGACTCCTTCAC-3' |
| HmiR-340-FO | F：5'-GCACGGTTATAAAGCAATGAGAC-3' |
| HmiR-340-RE | R：5'-TATGGTTGTTCTCGACTCCTTCAC-3' |
| HmiR-487b-3p-FO | F：5'-GATGCTCAAATCGTACAGGGTC-3' |
| HmiR-487b-3p-RE | R：5'-TATGGTTGTTCTGCTCTCTGTCTC-3' |
| HmiR-668-3p-FO | F：5'-ATTGTATGTCACTCGGCTCGG-3' |
| HmiR-668-3p-RE | R：5'-TATGGTTGTTCACGACTCCTTCAC-3' |
| HmiR-767-5p –FO-2 | F：5'-TGCTCATGCACCATGGTTG-3' |
| HmiR-767-5p –RE-2 | R：5'-TATGGTTGTTCACGACTCCTTCAC-3' |
| H-U6-FO | F：5'-ATTGGAACGATACAGAGAAGATT-3' |
| H-U6-RE | R：5'-GGAACGCTTCACGAATTTG-3' |
| F, forward; R, reverse; | |
