# Supplementary material for: High Expression MicroRNA-206 Inhibits the Growth of Tumor Cells in Human Malignant Fibrous Histiocytoma
Source: Front Cell Dev Biol. 2021 Nov 25;9:751833. doi: 10.3389/fcell.2021.751833 (PMC8656228; doi:10.3389/fcell.2021.751833)
Supplement: Supplementary file 2 [file Table_2.DOC]

Table II-A. Upregulation of miRNAs expression in malignant fibrous histiocytoma

| miRNA | P-value | GMI in ALDH^+^ | GMI in ALDH^-^ | Fold-change（+/-） |
| --- | --- | --- | --- | --- |
| hsa-miR-4780 | 0.0002758 | 17.69 | 4.05 | 4.36 |
| hsa-miR-4709-3p | 0.0010424 | 6.81 | 2.71 | 2.51 |
| hsa-miR-4804-3p | 0.0010717 | 4.29 | 1.61 | 2.67 |
| hsa-miR-4284 | 0.0011354 | 35.9 | 7.44 | 4.83 |
| hsa-miR-3191-5p | 0.0017497 | 2.75 | 1.37 | 2.01 |
| hsa-miR-668-3p | 0.0193703 | 3.81 | 1.8 | 2.11 |
| hsa-miR-183-3p | 0.0196483 | 9.43 | 2.02 | 4.67 |
| hsa-miR-4299 | 0.0218694 | 9.01 | 3.36 | 2.68 |
| hsa-miR-30e-3p | 0.0411931 | 4.38 | 1.89 | 2.31 |
| hsa-miR-381-5p | 0.0425425 | 5.32 | 1.55 | 3.44 |
| hsa-miR-3686 | 0.0454176 | 12.56 | 1.73 | 7.25 |
| hsa-miR-3136-3p | 0.0132262 | 3.33 | 1.57 | 2.12 |
| hsa-miR-4639-3p | 0.0133806 | 32.31 | 8.84 | 3.66 |
| hsa-miR-206 | 0.0149211 | 4.3 | 1.25 | 3.44 |
| hsa-miR-221-5p | 0.0401019 | 3.59 | 1.58 | 2.27 |
| hsa-miR-3646 | 0.040297 | 9.53 | 4.19 | 2.27 |

GMI in ALDH^+^：Geom mean of intensities in class ALDH^+^. GMI in ALDH^-^：Geom mean of intensities in class ALDH^-^.

Table II-B. Upregulation of miRNAs expression in malignant fibrous histiocytoma

| miRNA | P-value | GMI in ALDH^+^ | GMI in ALDH^-^ | Fold-change（+/-） |
| --- | --- | --- | --- | --- |
| hsa-miR-937-3p | 0.0204963 | 2.68 | 57.99 | 0.046 |
| hsa-miR-127-3p | 0.0159671 | 1.24 | 2.64 | 0.47 |
| hsa-miR-1275 | 0.0219473 | 3.85 | 25.36 | 0.15 |
| hsa-miR-3175 | 0.0226958 | 319.9 | 1169225239 | 0.00000027 |
| hsa-miR-5089-5p | 0.024819 | 7.16 | 61 | 0.12 |
| hsa-miR-550a-5p | 0.0295313 | 1.3 | 24.33 | 0.054 |
| hsa-miR-301a-5p | 0.029744 | 1.47 | 15.9 | 0.092 |
| hsa-miR-550b-2-5p | 0.0324293 | 1.55 | 33.16 | 0.047 |
| hsa-miR-3907 | 0.0327465 | 1.86 | 127.73 | 0.015 |
| hsa-miR-134-3p | 0.0346898 | 2.43 | 44.45 | 0.055 |
| hsa-miR-3156-3p | 0.0361597 | 2.62 | 5.48 | 0.48 |
| hsa-miR-340-5p | 0.0365038 | 8.48 | 8281.84 | 0.001 |
| hsa-miR-711 | 0.0365849 | 2.35 | 9.19 | 0.26 |
| hsa-miR-5196-3p | 0.0377247 | 1.81 | 4.4 | 0.41 |
| hsa-miR-30c-1-3p | 0.0389013 | 1.79 | 10.45 | 0.17 |
| hsa-miR-125b-1-3p | 0.0401197 | 1.46 | 32.91 | 0.044 |
| hsa-miR-4532 | 0.0401777 | 7.46 | 148.36 | 0.05 |
| hsa-miR-1290 | 0.0403046 | 2.45 | 14.49 | 0.17 |
| hsa-miR-5581-3p | 0.0407419 | 607.84 | 1294055315 | 0.00000047 |
| hsa-miR-4686 | 0.0461934 | 1.29 | 3.73 | 0.35 |
| hsa-miR-4447 | 0.0483457 | 16.66 | 582.92 | 0.029 |
| hsa-miR-767-5p | 0.0485028 | 645.76 | 441891276.8 | 0.0000015 |
| hsa-miR-4524b-5p | 0.0488747 | 1.32 | 6.51 | 0.2 |
| hsa-miR-1264 | 0.0494311 | 5.31 | 28.91 | 0.18 |
| hsa-miR-550a(-3)-5p | 0.0455415 | 12.48 | 13669.22 | 0.00091 |
| hsa-miR-138-2-3p | 0.001872 | 2.65 | 22.72 | 0.12 |
| hsa-miR-4482-3p | 0.0040754 | 1.25 | 3.03 | 0.41 |
| hsa-miR-519e-3p | 0.0070972 | 1.73 | 54.69 | 0.032 |
| hsa-miR-302e | 0.0086638 | 3.13 | 12.78 | 0.24 |
| hsa-miR-99b-3p | 0.0088791 | 1.77 | 3.93 | 0.45 |
| hsa-miR-5580-5p | 0.0123728 | 1.55 | 4.32 | 0.36 |

GMI in ALDH^+^：Geom mean of intensities in class ALDH^+^. GMI in ALDH^-^: Geom mean of intensities in class ALDH^-^
