# Supplementary material for: High Expression MicroRNA-206 Inhibits the Growth of Tumor Cells in Human Malignant Fibrous Histiocytoma
Source: Front Cell Dev Biol. 2021 Nov 25;9:751833. doi: 10.3389/fcell.2021.751833 (PMC8656228; doi:10.3389/fcell.2021.751833)
Supplement: Supplementary file 3 [file Table_3.DOC]

**Table III-A. The number of target genes predicted by Upregulated miRNAs**

| Upregulated miRNA | Gene predicted |
| --- | --- |
| hsa-miR-4780 | 45 |
| hsa-miR-4709-3p | 145 |
| hsa-miR-4804-3p | 21 |
| hsa-miR-4284 | 49 |
| hsa-miR-3191-5p | 149 |
| hsa-miR-668-3p | 46 |
| hsa-miR-183-3p | 76 |
| hsa-miR-4299 | 50 |
| hsa-miR-30e-3p | 264 |
| hsa-miR-381-5p | 4 |
| hsa-miR-3686 | 212 |
| hsa-miR-3136-3p | 43 |
| hsa-miR-4639-3p | 30 |
| hsa-miR-206 | 219 |
| hsa-miR-221-5p | 141 |
| hsa-miR-3646 | 392 |

**Table III-B The number of target genes predicted by Downregulated miRNAs**

| Downregulated miRNA | Gene predicted |
| --- | --- |
| hsa-miR-937-3p | 1 |
| hsa-miR-127-3p | 3 |
| hsa-miR-1275 | 61 |
| hsa-miR-3175 | 133 |
| hsa-miR-5089-5p | 62 |
| hsa-miR-550a-5p | 89 |
| hsa-miR-301a-5p | 76 |
| hsa-miR-550b-2-5p | 79 |
| hsa-miR-3907 | 48 |
| hsa-miR-134-3p | 63 |
| hsa-miR-3156-3p | 69 |
| hsa-miR-340-5p | 297 |
| hsa-miR-711 | 55 |
| hsa-miR-5196-3p | 29 |
| hsa-miR-30c-1-3p | 124 |
| hsa-miR-125b-1-3p | 1 |
| hsa-miR-4532 | 8 |
| hsa-miR-1290 | 90 |
| hsa-miR-5581-3p | 68 |
| hsa-miR-4686 | 86 |
| hsa-miR-4447 | 39 |
| hsa-miR-767-5p | 190 |
| hsa-miR-4524b-5p | 201 |
| hsa-miR-1264 | 136 |
| hsa-miR-550a(-3)-5p | 65 |
| hsa-miR-138-2-3p | 76 |
| hsa-miR-4482-3p | 219 |
| hsa-miR-519e-3p | 88 |
| hsa-miR-302e | 121 |
| hsa-miR-99b-3p | 8 |
| hsa-miR-5580-5p | 118 |
