# Supplementary material for: High Expression MicroRNA-206 Inhibits the Growth of Tumor Cells in Human Malignant Fibrous Histiocytoma
Source: Front Cell Dev Biol. 2021 Nov 25;9:751833. doi: 10.3389/fcell.2021.751833 (PMC8656228; doi:10.3389/fcell.2021.751833)
Supplement: Supplementary file 4 [file Table_4.DOC]

**Table IV The significant gene functions screened by experiment**

| Significant gene function | gene count |
| --- | --- |
| **up-regulated** |  |
| transcription, DNA-dependent | 194 |
| regulation of transcription, DNA-dependent | 132 |
| signal transduction | 128 |
| positive regulation of transcription from RNA polymerase II promoter | 91 |
| small molecule metabolic process | 88 |
| **down-regulated** |  |
| transcription, DNA-dependent | 257 |
| regulation of transcription, DNA-dependent | 162 |
| small molecule metabolic process | 143 |
| positive regulation of transcription from RNA polymerase II promoter | 137 |
| signal transduction | 134 |
| positive regulation of transcription, DNA-dependentr | 98 |
