# Supplementary material for: High Expression MicroRNA-206 Inhibits the Growth of Tumor Cells in Human Malignant Fibrous Histiocytoma
Source: Front Cell Dev Biol. 2021 Nov 25;9:751833. doi: 10.3389/fcell.2021.751833 (PMC8656228; doi:10.3389/fcell.2021.751833)
Supplement: Supplementary file 5 [file Table_5.DOC]

Table V-A: The significant miRNAs screened by experiment （ Degree＞68）

| MicroRNA | style | Degree |
| --- | --- | --- |
| hsa-miR-340-5p | down | 90 |
| hsa-miR-4524b-5p | down | 87 |
| hsa-miR-4482-3p | down | 87 |
| hsa-miR-767-5p | down | 83 |
| hsa-miR-302e | down | 78 |
| hsa-miR-3646 | up | 77 |
| hsa-miR-3191-5p | up | 74 |
| hsa-miR-30e-3p | up | 73 |
| hsa-miR-5089-5p | down | 72 |
| hsa-miR-3175 | down | 69 |
| hsa-miR-206 | up | 69 |
| hsa-miR-30c-1-3p | down | 68 |

| Path name | Degree |
| --- | --- |
| Metabolic pathways | 40 |
| MAPK signaling pathway | 39 |
| Pathways in cancer | 38 |
| Regulation of actin cytoskeleton | 37 |
| Proteoglycans in cancer | 37 |
| HTLV-I infection | 36 |
| Endocytosis | 35 |
| Focal adhesion | 34 |
| Hepatitis B | 34 |
| Wnt signaling pathway | 34 |
| TGF-beta signaling pathway | 32 |
| Calcium signaling pathway | 32 |

Table V-B: The significant pathways screened by experiment （ Degree＞31）
